# An attribute control chart for a Weibull distribution under accelerated hybrid censoring

**DOI:** 10.1371/journal.pone.0173406

**Published:** 2017-03-03

**Authors:** Muhammad Aslam, Osama H. Arif, Chi-Hyuck Jun

**Affiliations:** 1 Department of Statistics, Faculty of Science, King Abdulaziz University, Jeddah, Saudi Arabia; 2 Department of Industrial and Management Engineering, POSTECH, Pohang, Republic of Korea; University of Rijeka, CROATIA

## Abstract

In this article, an attribute control chart has been proposed using the accelerated hybrid censoring logic for the monitoring of defective items whose life follows a Weibull distribution. The product can be tested by introducing the acceleration factor based on different pressurized conditions such as stress, load, strain, temperature, etc. The control limits are derived based on the binomial distribution, but the fraction defective is expressed only through the shape parameter, the acceleration factor and the test duration constant. Tables of the average run lengths have been generated for different process parameters to assess the performance of the proposed control chart. Simulation studies have been performed for the practical use, where the proposed chart is compared with the Shewhart np chart for demonstration of the detection power of a process shift.

## Introduction

Control charts have been developed for monitoring the production process for any unusual change in the product [1–3]. Since its inception by Walter A. Shewhart during 1920’s, it has been applied to many disciplines for quality improvement including health care, nuclear engineering, analytic laboratories, education, etc. Two types of control charts have been developed: variable charts are used when the quality characteristic of interest is measureable as length, weight, etc. and the attribute charts are available in the literature when the quality characteristic is non-measureable as good/defective, yes/no, etc.

Hybrid censoring scheme is commonly used in the life testing situations which was introduced by Epstein [1] for the exponential distribution as the life time distribution. Later on [2] probed the hybrid censoring scheme and proposed confidence intervals. In hybrid censoring both the time and the number of failures are considered for the life testing of the product. Gupta and Kundu [3] developed the hybrid censoring for the exponential failure distribution. There are situations in which only the time is fixed for the life testing, which is known as the Type-I censoring. When the number of observed failures is fixed in the life testing, it is called the Type-II censoring. The combination of these two censoring schemes is called the hybrid censoring [4]. The hybrid censoring has been studied by many authors including [5–8] and [9].

Product reliability plays a prime role in the success of the production process. Often, the quality control personnel use the life-testing technique for the reliability of the products, using few units. Product accelerated life-testing ensures the use of the product in different pressurized/stress situations like temperature, pressure, load, voltage etc. In this era of most modern technology, most of the goods are manufactured with a very high product reliability and the life-testing of such products require much time and cost [10]. Accelerated life testing technique is employed to experimental products with varying accelerated factors for its possible quick failure (declared as defective in our case) than in normal use to notice its failures [4]. The statistical models for reliability data have been thoroughly discussed by Meeker and Escobar [11], [12]. The accelerated life testing methodology have been studied by many researchers including [13, 14].

In recent years, several useful methods have been developed for the accelerated lifetime testing of the products. Motivated with these methods, the attribute accelerated life testing control chart has been developed in this article. To the best of the researcher’s knowledge, no control chart has been developed using the accelerated life testing for the attribute data.

## Design of proposed control chart

We propose the following np control chart for the Weibull distribution under the accelerated hybrid censoring test:

**Step-1:** Select a random sample of size *n* from the production process and put them on the accelerated hybrid censoring test subject to the accelerated condition with censoring time *τ*_*A*_ when acceleration factor (AF) is known. Count the number of failed items by time *τ*_*A*_, denoted by *D*.

**Step-2:** Declare the process as out-of-control if *D* ≥ *UCL* or *D* ≥ *LCL*. Otherwise (if LCL < D < UCL), declare the process as in-control.

It is assumed that the lifetime of the product under the use condition, denoted by *T*_*U*_, follows the Weibull distribution with shape parameter *γ* and scale parameter *φ*_*U*._ The cumulative distribution function (cdf) of the Weibull distribution is given as

$$F_{U}\left(t_{U}\right)=1-exp\left[-{\left(t_{U}/\varphi_{U}\right)}^{\gamma }\right]$$ (1)

On the other hand, the lifetime under the acceleration condition is assumed to follow the Weibull distribution with the cdf of

$$F_{\text{A}}\left(t_{\text{A}}\right)=1-exp\left[-{\left(t_{A}/\varphi_{A}\right)}^{\gamma }\right]$$ (2)

where *φ*_A_ is the scale parameter under the acceleration condition. The average lifetime of the product under the acceleration condition is given by

$$\mu_{A}=\left(\varphi_{A}/\gamma \right)\Gamma \left(1/\gamma \right)$$ (3)

Let us assume that

$$\varphi_{A}=\varphi_{\text{U}}/AF$$ (4)

Then, Eq (2) can be written as follows

$$F_{\text{A}}\left(t_{\text{A}}\right)=1-exp\left[-{\left(t_{A}\times AF/\varphi_{U}\right)}^{\gamma }\right]$$ (5)

The chance that a unit on test fails until the censoring time *τ*_*A*_ at the accelerated condition during the in-control process is given as

$${\text{p}}_{0}=1-exp\left[-{\left(\tau_{A}\times AF/\varphi_{U}\right)}^{\gamma }\right]$$ (6)

It is convenient to specify the censoring time as a fraction of mean lifetime. So, let

$$\tau_{A}=a\mu_{A}=a\left(\varphi_{A}/\gamma \right)\Gamma \left(1/\gamma \right)$$

where *a* is the test duration constant between 0 and 1. Then, Eq (6) is rewritten by

$${\text{p}}_{0}=1-\text{ exp}\left[-a^{\gamma }{\left(AF\right)}^{\gamma }{\left(\Gamma \left(1/\gamma \right)/\gamma \right)}^{\gamma }\right]$$ (7)

Hence, the number of failed items by time *τ*_*A*_ follows a binomial distribution with parameters n and p_0_ if each item may fail independently. Therefore, two control limits for the proposed np control chart are constructed as

$$UCL=np_{0}+k\sqrt{n{\text{p}}_{0}\left(1-{\text{p}}_{0}\right)}$$ (8)

$$LCL=max[0,n{\text{p}}_{0}-k\sqrt{n{\text{p}}_{0}\left(1-{\text{p}}_{0}\right)}]$$ (9)

where *k* is the control constant to be determined by considering the in-control average run length (ARL).

The probability that the process is declared as in control at acceleration condition is given as

$$P_{in}^{0}=P\left\{LCL\leq D\leq UCL|{\text{p}}_{0}\right\}$$ (10)

$$P_{in}^{0}=\sum_{d=\left\lfloor LCL\right\rfloor +1}^{\left\lfloor UCL\right\rfloor }\left(\begin{array}{c} n\\ d \end{array}\right){\left(p_{0}\right)}^{d}{\left(1-{\text{p}}_{0}\right)}^{n-d}$$ (11)

The ARL for the in-control process is obtained by

$$ARL_{0}=\frac{1}{1-P_{in}^{0}}$$ (12)

The proposed control chart involves design parameters of sample size (n), test duration constant (*a*) and control constant (*k*). In this study, the sample size is assumed to be specified and the constants *a* and *k* will be determined so that *ARL*_0_ equals the target value r_0_.

## Under shifted process

Here it is assumed that the process may shift, in which case the lifetime follows the Weibull distribution having a new scale parameter of *cφ*_*U*_ with the shift constant c while having the same shape parameter.

So, the chance that a unit on test fails until the censoring time at accelerated condition after process shift is given as

$$p_{1}=1-exp\left[-{\left(\tau_{A}\times AF/c\varphi_{\text{U}}\right)}^{\gamma }\right]$$ (13)

It is rewritten by

$${\text{p}}_{1}=\;1-\text{exp}\left[-a^{\gamma }{\left(AF\right)}^{\gamma }{\left(\Gamma \left(1/\gamma \right)/c\gamma \right)}^{\gamma }\right]$$ (14)

The probability that the process is declared as in control at acceleration condition after the process shift is given as

$$P_{in}^{1}=P\left\{LCL\leq D\leq UCL|{\text{p}}_{1}\right\}$$ (15)

$$P_{in}^{1}=\sum_{d=\left\lfloor LCL\right\rfloor +1}^{\left\lfloor UCL\right\rfloor }\left(\begin{array}{c} n\\ d \end{array}\right){\left(p_{1}\right)}^{d}{\left(p_{1}\right)}^{n-d}$$ (16)

The ARL for the out-of-control process is

$$ARL_{1}=\frac{1}{1-P_{in}^{1}}$$ (17)

Using the above mentioned equations a coding program was written in R-language to estimate the design parameters (Coding program can be obtained on request). Tables 1–3 have been generated for r_0_ = 370 and 300, n = 30, γ = 2 and 3. The accelerated factor (AF) has been determined using the accelerated factor models described in Aslam, Jun [15]. The average run length (ARL) is the commonly used measure for the evaluation of the proposed control chart in the area of quality control charts. The ARL may be defined as the average number of samples to be plotted before the process indicates an out-of-control signal [16]. The ARL_1_ values have been generated using different levels c = 1, 0.99, 0.95, 0.93, 0.91, 0.90, 0.88, 0.85, 0.80, 0.75, 0.70, 0.60, 0.50, 0.40, 0.30, 0.20 and 0.10. The ARL as a performance measure has been studied by many researchers including [17–20].

**Table 1:** The values of ARL when *r*_0_ = 370;*n* = 30;γ = 2.

AF	6	7.623	8.52	9	12.9	14	20	20.09	24.5	26
a	0.0689	0.1148	0.0486	0.0787	0.0410	0.0598	0.0167	0.0166	0.0169	0.0247
k	3.0865	3.0682	3.0085	3.0181	3.0777	3.0023	3.0528	3.0042	3.0758	3.0985
c	ARLs									
1.00	370.29	370.48	370.18	370.08	370.72	370.01	370.16	370.01	371.71	370.48
0.99	321.90	342.34	321.80	335.60	313.93	324.77	328.25	328.11	323.13	308.77
0.95	183.48	179.52	183.43	182.17	161.78	156.77	202.16	202.08	184.16	147.58
0.93	138.43	118.71	138.39	125.60	116.39	103.79	158.31	158.25	138.93	102.14
0.91	104.44	77.60	104.41	85.53	83.92	68.52	123.82	123.77	104.81	70.96
0.90	90.72	62.78	90.70	70.52	71.33	55.78	109.46	109.42	91.04	59.26
0.88	68.49	41.33	68.47	48.06	51.67	37.22	85.49	85.46	68.72	41.51
0.85	45.02	22.58	45.01	27.40	32.09	20.74	58.95	58.92	45.16	24.68
0.80	22.60	8.95	22.59	11.36	14.94	8.46	31.71	31.70	22.67	10.89
0.75	11.60	4.04	11.60	5.18	7.32	3.92	17.16	17.16	11.63	5.21
0.70	6.19	2.16	6.19	2.69	3.86	2.13	9.43	9.43	6.20	2.78
0.60	2.15	1.11	2.15	1.20	1.50	1.11	3.16	3.16	2.15	1.24
0.50	1.16	1.00	1.16	1.00	1.03	1.00	1.42	1.42	1.16	1.01
0.40	1.00	1.00	1.00	1.00	1.00	1.00	1.03	1.03	1.00	1.00
0.30	1.00	1.00	1.00	1.00	1.00	1.00	1.00	1.00	1.00	1.00
0.20	1.00	1.00	1.00	1.00	1.00	1.00	1.00	1.00	1.00	1.00
0.10	1.00	1.00	1.00	1.00	1.00	1.00	1.00	1.00	1.00	1.00

**Table 2:** The values of ARL when *r*_*0*_ = 300;*n* = 30;γ = 2.

AF	6	7.623	8.52	9	12.9	14	20	20.09	24.5	26
a	0.0700	0.0652	0.0300	0.0182	0.0415	0.0213	0.0229	0.0359	0.0155	0.0115
k	3.1456	3.1398	3.0601	3.0660	3.0365	3.0013	3.0613	3.0079	3.1479	3.0608
c	ARLs									
1.00	300.10	300.21	300.09	300.71	300.04	300.52	300.89	301.61	300.28	301.37
0.99	261.35	256.90	272.43	280.13	254.67	269.72	259.68	261.82	263.99	270.48
0.95	150.11	137.94	184.12	209.85	132.56	174.25	144.06	131.16	157.30	174.72
0.93	113.73	101.23	150.92	181.04	95.88	139.70	107.36	90.03	121.28	140.08
0.91	86.18	74.42	123.47	155.83	69.53	111.84	80.09	61.67	93.46	112.14
0.90	75.04	63.86	111.60	144.46	59.28	100.01	69.20	51.10	82.04	100.28
0.88	56.92	47.12	91.06	123.92	43.20	79.91	51.74	35.26	63.22	80.11
0.85	37.71	30.05	66.90	98.03	27.11	56.95	33.60	20.55	42.81	57.10
0.80	19.21	14.55	39.74	65.57	12.85	32.28	16.65	8.89	22.49	32.36
0.75	10.03	7.36	23.46	43.21	6.43	18.30	8.54	4.25	12.01	18.34
0.70	5.46	3.97	13.83	28.08	3.48	10.46	4.62	2.33	6.60	10.48
0.60	1.99	1.56	4.94	11.42	1.43	3.66	1.74	1.15	2.34	3.66
0.50	1.13	1.04	2.02	4.55	1.03	1.60	1.08	1.00	1.21	1.60
0.40	1.00	1.00	1.15	1.95	1.00	1.06	1.00	1.00	1.01	1.06
0.30	1.00	1.00	1.00	1.11	1.00	1.00	1.00	1.00	1.00	1.00
0.20	1.00	1.00	1.00	1.00	1.00	1.00	1.00	1.00	1.00	1.00
0.10	1.00	1.00	1.00	1.00	1.00	1.00	1.00	1.00	1.00	1.00

**Table 3:** The values of ARL when *r*_*0*_ = 370;*n* = 30;γ = 3.

AF	6	7.623	8.52	9	12.9	14	20	20.09	24.5	26
a	0.1530	0.0928	0.0903	0.0456	0.0416	0.0293	0.0268	0.0336	0.0276	0.0221
k	3.0420	3.0110	3.1161	3.0970	3.0686	3.1430	3.1089	3.0090	3.1390	3.0415
c	ARLs									
1.00	370.25	370.18	370.00	370.52	370.59	370.92	370.66	370.10	370.76	370.35
0.99	301.42	285.27	281.53	320.08	304.86	320.42	304.92	288.61	289.11	300.31
0.95	95.50	102.94	94.73	177.50	140.15	177.69	140.17	108.74	108.91	130.76
0.93	52.37	62.90	55.95	131.92	95.38	132.06	95.39	67.69	67.80	86.81
0.91	29.43	38.99	33.66	97.97	65.15	98.07	65.17	42.65	42.72	57.96
0.90	22.32	30.88	26.31	84.41	53.95	84.49	53.95	34.03	34.08	47.48
0.88	13.20	19.65	16.38	62.66	37.14	62.72	37.15	21.92	21.95	32.06
0.85	6.48	10.38	8.46	40.12	21.51	40.15	21.51	11.74	11.75	18.12
0.80	2.52	4.10	3.34	19.25	9.11	19.26	9.11	4.66	4.66	7.48
0.75	1.39	2.01	1.71	9.46	4.23	9.47	4.23	2.24	2.24	3.48
0.70	1.07	1.28	1.17	4.87	2.25	4.88	2.25	1.37	1.37	1.90
0.60	1.00	1.00	1.00	1.69	1.11	1.69	1.11	1.01	1.01	1.05
0.50	1.00	1.00	1.00	1.04	1.00	1.04	1.00	1.00	1.00	1.00
0.40	1.00	1.00	1.00	1.00	1.00	1.00	1.00	1.00	1.00	1.00
0.30	1.00	1.00	1.00	1.00	1.00	1.00	1.00	1.00	1.00	1.00
0.20	1.00	1.00	1.00	1.00	1.00	1.00	1.00	1.00	1.00	1.00
0.10	1.00	1.00	1.00	1.00	1.00	1.00	1.00	1.00	1.00	1.00

From Tables 1–3, we note the following trends in control chart parameters.

1. For the same values of other parameters, we note a decreasing trend in *ARL*_1_ as *γ* increases from 2 to 3.
2. For the same values of other parameters, *ARL*_1_ decreases as AF increases.
3. For the same values of other parameters, *ARL*_1_ decreases as *ARL*_0_ increases.

## Models of AF

Different values of the AF including 6.00, 7.623, 8.52, 9.00, 12.90, 14.00, 20.00, 20.09, 24.50 and 26.00 have been used to explain the proposed control chart. The first value of AF = 6.00 has been used with the methodology in [21] for the calculation of AF. The acceleration of unreliability as a function of junction temperature and power can be described as

$$AF\propto P^{n}\text{exp}\left(-E_{a}/\left(K_{B}.T_{j}\right)\right)$$

where P is the power, n is the acceleration parameter of the power, *E*_*a*_ is the activation energy, *K*_*B*_ is the Boltzmann’s constant and *T*_*j*_ is the junction temperature. The value of *E*_*a*_ ranges from 0.41 eV to 0.64 eV and n ranges from 2.2 to 5.9. Using the 90% confidence level, the acceleration parameters *n* > 2.7 and *E*_*a*_ > 0.64 have been extrapolated using the exponential distribution. The AF = 6 in a multi- cell life test has been extrapolated for P = 13.6 with junction temperature = 64. More details can be seen in [21].

The second value of AF = 7.623 has been used in [22], where the AF value has been proposed for the thermal stress and operating which can be describes as

$$\text{AF}=e^{\frac{E_{a}}{K}\left(\frac{1}{T_{0}}-\;\frac{1}{T_{s}}\right)}$$

Where *E*_*a*_ is the activation energy equal to 0.3 eV, K = 8.617x10^-5^ = 1/11, Boltzmann’s constant 605 eV/k°, the operating temperature, T_0_ = 50°C and the stress temperature, T_s_ = 125°C. More details can be seen in [22].

The third value of AF = 8.52 has been used in [23], where the AF value has been proposed for the thermal use and stress which can be described as

$$\text{AF }=e^{\frac{E_{a}}{K}\left(\frac{1}{T_{use}}-\;\frac{1}{T_{stress}}\right)}$$

where *E*_*a*_ is the thermal activation energy and is equal to 0.3 eV, K is the Boltzmann’s constant and is equal to 8.63x10^-5^ eV/K, *T*_*use*_ is the use temperature and is equal to 273+C° degree Kelvin and *T*_*stress*_ is the stress temperature in Kelvin degree of the life test and is equal to 273+C°. More details can be seen in [23].

The calculation details of the other AF values of 9.00, 12.90, 14.00, 20.00, 20.09, 24.50 and 26.00 can be seen in [15].

## Application to a semiconductor device case

In this section, an application of the proposed control chart is given for the monitoring of the thermal acceleration of semiconductor device failure mechanisms. The life test of the thermal stress and operating condition has been explained for physio-chemical reaction rates [23]. Suppose that the proposed control chart is used, where the censoring time at the accelerated condition is 500h, while other parameters are set as n = 30,AF = 7.623, and r_0_ = 370. It is known that the device failure time follows a Weibull distribution with γ = 2. From Table 1 we select the design parameters of the proposed chart as *a* = 0.1148, *k* = 3.0682. The value of *a* = 0.1148 indicates that the censoring time of 500h is just 11.5% of the mean failure time of the device. From Eq(7), the probability that an item fails before the censoring time is obtained by *p*_*0*_ = 0.4520. Also, UCL and LCL are calculated from Eqs (8) and (9) by UCL = 24, LCL = 8. Considering these parameters, the proposed control chart can be used by applying the same steps given above.

**Fig 1:**
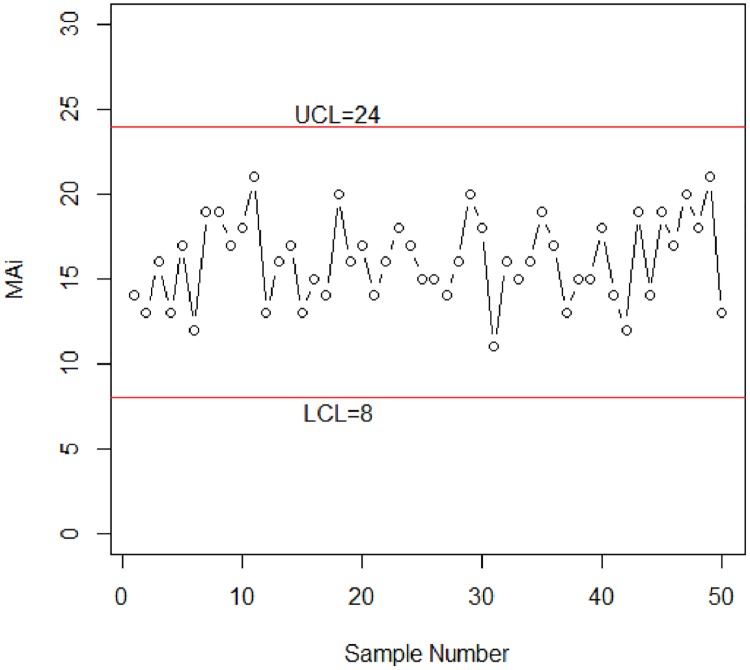
Control Chart for semiconductor device case.

Suppose now that 50 observations (data of D’s—each observation corresponds to an independent test to count the number of failures from 30 items) have been generated from a binomial distribution with n = 30 and *p*_*0*_ = 0.4520 as in Table 4. Fig 1 plots the proposed control chart with the upper control limit (UCL) = 24 and the lower control limit (LCL) = 8.

**Table 4:** Data for semiconductor device case.

14	13	16	13	17	12	19	19	17	18
21	13	16	17	13	15	14	20	16	17
14	16	18	17	15	15	14	16	20	18
11	16	15	16	19	17	13	15	15	18
14	12	19	14	19	17	20	18	21	13

It is seen from Fig 1 that no observation shows an unusual pattern, which means that the process is in control.

## Simulation study

In this section we demonstrate the efficiency of the proposed control chart in quick detection of the out-of-control process through a simulation data. Table 5 shows the 50 observations (data of D’s) generated with the design parameters of γ = 2, AF = 7.623, and *a* = 0.1188.

**Table 5:** Simulation data with *λ* = 2, *a* = 0.1188, AF = 7.623 and *c* = 0.85.

21	10	18	16	14	14	17	16	18	15
18	19	14	18	17	19	19	14	18	15
16	11	13	13	13	14	14	15	16	19
12	14	17	15	11	18	10	13	6	16
17	11	15	14	14	11	16	12	10	7

**Fig 2:**
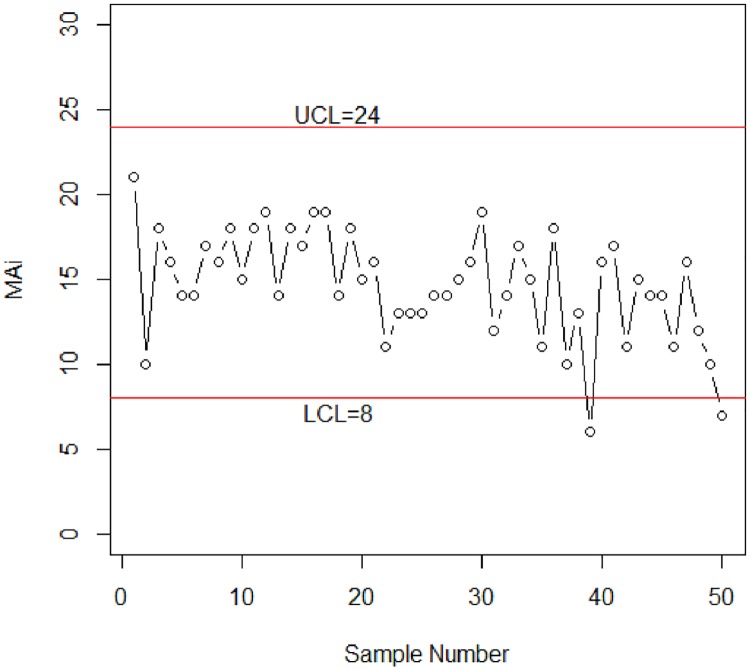
Control chart for simulation data.

The first 20 observations are generated from the in-control process (that is, from a binomial distribution with n = 30 and p_*0*_ = 0.54799 and the next 30 observations are generated from the shifted process using the shifted scale parameter *φ*_U1_ = *cφ*_*U*_, where *c* is 0.85. So, the 30 observations are generated from a binomial distribution with n = 30 and *p*_*1*_ = 0.43495. Fig 2 shows the proposed control chart with r_o_ = 370.

It can be seen from Fig 2 that the proposed control chart is detecting the out-of-control process at the 43^rd^ subgroup, which is 23^rd^ subgroup after the actual process shift.

**Fig 3:**
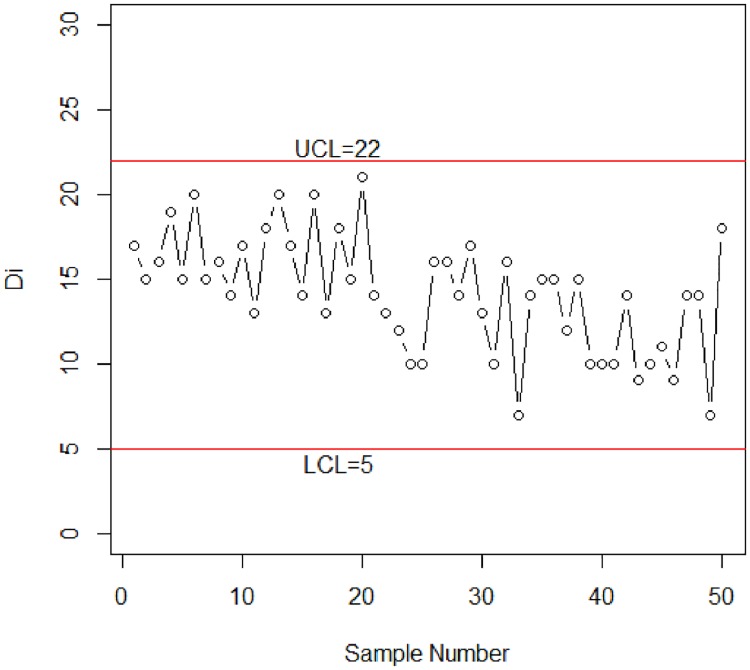
Shewhart-np control chart for simulation data.

To compare the performance of the proposed control chart with the existing one, the values of D are also plotted to the Shewhart-np control chart in Fig 3. The calculated control limits for this chart are shown in Fig 3 using $\bar{p}=\sum D/n=0.472$.

From Fig 3, it can be noted that all plotted values of D are within the UCL and LCL. So, the Shewhart-np control chart is unable to detect the shift in the process.

## Conclusions

In this article, an attribute control chart has been proposed using the accelerated hybrid censoring scheme under the Weibull life time distribution. The proposed control chart parameters have been estimated using different accelerated factors for different process settings. The comparative performance of the chart has been evaluated using the average run lengths of the in-control and the out-of-control processes. The proposed chart is shown to be better than the Shewhart-np control chart in detecting a shift in the process. The proposed chart can be extended to a variables control chart and/or other lifetime distributions including three parameters Weibull distribution as future research.

## Supporting information

S1 Data Data for semiconductor device case. (DOCX)
