# Supplementary material for: An attribute control chart for a Weibull distribution under accelerated hybrid censoring
Source: PLoS One. 2017 Mar 3;12(3):e0173406. doi: 10.1371/journal.pone.0173406 (PMC5336291; doi:10.1371/journal.pone.0173406)
Supplement: S1 Data — (DOCX) [file pone.0173406.s001.docx]

**Table 4: Data for semiconductor device case**

| 14 | 13 | 16 | 13 | 17 | 12 | 19 | 19 | 17 | 18 |
| --- | --- | --- | --- | --- | --- | --- | --- | --- | --- |
| 21 | 13 | 16 | 17 | 13 | 15 | 14 | 20 | 16 | 17 |
| 14 | 16 | 18 | 17 | 15 | 15 | 14 | 16 | 20 | 18 |
| 11 | 16 | 15 | 16 | 19 | 17 | 13 | 15 | 15 | 18 |
| 14 | 12 | 19 | 14 | 19 | 17 | 20 | 18 | 21 | 13 |
